# A History of Malaria and Conflict

**DOI:** 10.1007/s00436-024-08167-4

**Published:** 2024-03-20

**Authors:** Jonas E. Mertens

**Affiliations:** Institute for Infection Research and Vaccine Development (IIRVD), University Medical Center Hamburg-Eppendorf, Hamburg, Germany

**Keywords:** Malaria, War, *Plasmodium*, Military history, Infectious disease, Conflict, Refugees, Poverty, Eradication

## Abstract

It is supposed that in all armed conflicts until World War II more humans died of infectious diseases than of the actual violence. Especially malaria left a crucial imprint on wars throughout history. The disease aggravates wartime conditions, is thus responsible for significant morbidity and mortality in conflict zones, and is at the same time more commonly found in these areas. Malaria has halted many military campaigns in the past, with prominent examples ranging from antiquity through the medieval period and into the modern era. The parasitosis still continues to play an important role in the outcome of warfare and follow-up events today and is of special public health importance in areas of the Global South, where most of its endemicity and some of the most brutal conflicts of our time are located. Vice versa, wars and ensuing population movements increase malaria transmission and morbidity as well as impede control efforts. Awareness of this and the development of strategies to overcome both malaria and wars will massively improve the well-being of the population affected.

## Introduction

The history of humanity has been without a doubt distinctly shaped by wars, which may be defined as deliberate actions by organized groups against other groups involving the potential or actual use of (deadly) force (Ferguson 1984). Outcome of these depends on a large variety of factors, among them diseases that run rampant within the opposing factions. Especially infectious diseases play a substantial role, capitalizing on the conditions found during wars both in the armed forces as well as in civilians—poor sanitation, hygiene, and nutrition, lack of water, shelter, and health services as well as (man-made) ecological changes and population movements all contributing massively to their spread (Connolly and Heymann 2002; Shah 2010a).

Malaria has been described as “most sensitive to the relationship of human populations to their environment [of all high-impact infectious diseases]”, with no enterprise disrupting this relationship as profoundly as warfare does (Snowden 2006a). As the most pernicious parasitic illness in the world, it has just as well been closely intertwined with our history, not only impinging upon military actions, but also hindering settlement, political and economic efforts, particularly in the Global South, at the same time contributing to socioeconomic, gender, and racial inequalities (Athni et al. 2021). Humanity’s long past with the disease has caused genetic as well as cultural adaptations to occur for protection against infection.^1^ Co-evolution of the causative parasites, apicomplexans of the genus *Plasmodium*, and its hominid host has occurred since prehistoric times, with malaria taking a toll on human survival at least since the beginning of the Neolithic revolution (Harper and Armelagos 2010) or even earlier, i.e. since the Palaeolithic Era (Athni et al. 2021). The disease thus accounts by far for most estimated annual disability-adjusted life years (DALYs) among the vector-borne diseases today, with its dynamics changing and being changed by human behavior (ibid.).

The *Anopheles* mosquito serves as vector for *Plasmodium*, with the relevant species for infection in humans being *P. falciparum*, *P. vivax*, *P. ovale*, *P. malariae*, and *P. knowlesi.* Of these, *P. falciparum* regularly induces the most severe of clinical presentations and today is responsible for the bulk of global morbidity and mortality, especially in sub-Saharan Africa, while the disease as a whole is found in the tropics and subtropics on both sides of the equator (World Health Organization 2023a). Outside of Africa, the majority of cases is caused by *P. vivax* (ibid.), with the differing distribution relying on a multitude of factors (Price et al. 2020), among them *P*. *vivax*’ ability to stay dormant within the patient’s liver, forming hypnozoites, which may facilitate a recurrence of clinical disease even years after the initial infection (White 2011), rendering vector control measures less effective.

Typical symptoms of infection include intermittent fevers, fatigue, nausea, headaches, and organ swelling; in severe malaria, coma and death can occur, especially in children and pregnant women. Treatment usually involves the use of antimalarials (e.g. an artemisinin-based combination therapy); preventive measures against mosquito bites (e.g. insecticide-treated bed nets) and aforementioned vector control procedures are just as important in controlling the disease worldwide. Unfortunately, by now, resistances against all antimalarials as well as most insecticides have emerged (Hemingway et al. 2016; Menard and Dondorp 2017), particularly relevant in *P. falciparum.*

**Fig. 1:**
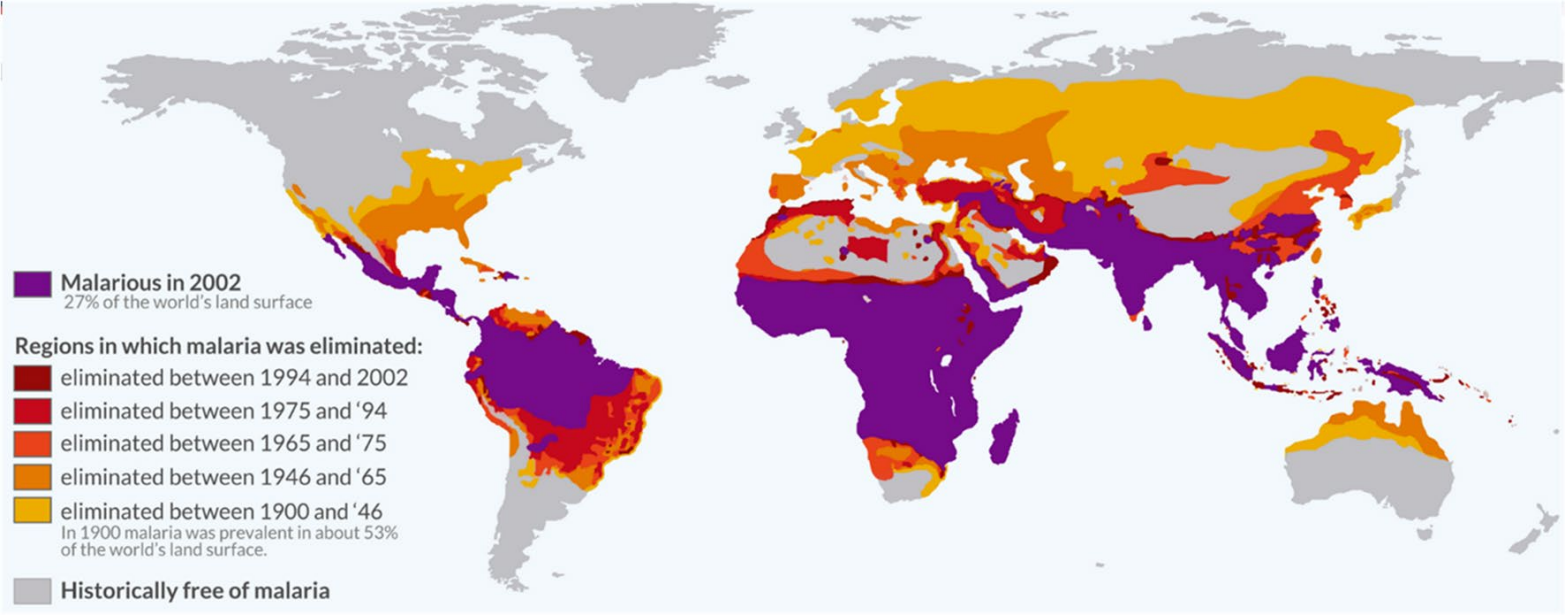
Malaria prevalence throughout the twentieth century and malaria-endemic countries in 2002. The disease’s endemicity was at its maximum around 1900, extending from the 64th parallel north, incorporating temperate regions of Europe, to the 32nd parallel south, corresponding to 15° C July and January isotherms (Hay et al. 2004). From then on, malaria prevalence regressed further and further toward the equator. This figure is an adaptation of the original image published online at OurWorldInData.org (Roser and Ritchie 2022), which was adapted in turn from a figure found in Hay et al. (2004)

Looking at the disease from a historical point of view, the endemicity of malaria was much more extensive, incorporating also more temperate regions, in Europe extending North into several parts of England^2^ (Dobson 1994; Reiter 2000), being an important cause of morbidity and mortality until the nineteenth century; and even Southern Finland and Scandinavia (Southern Norway, Central Sweden) as well as some parts of Russia until the twentieth century (Huldén et al. 2005; Piperaki 2018) (Fig. 1).

It is already well known and summarized (Kakkilaya 2015) that malaria has had an impact on conflicts and the outcomes of warfare in its—formerly much larger—regions of endemicity throughout history. In 2007, medical historian Randall M. Packard noted that if one would overlay the countries with high malaria incidence with a world map of armed conflicts or distributions of refugee populations, they would match quite closely (Packard 2007a), something that still holds true to a certain degree today (Messenger et al. 2021). The list of potential conflicts in which the disease has supposedly had an influence on the outcome is a long one, and this article shall shed light on the most prominent of these, broadly subdivided by era they occurred in.

It must be mentioned however, that just as the distribution of malaria has changed over the centuries, so have armed conflicts: The ends, military techniques, armies, and economy involved all differ in pre-modern, modern, and post-modern varieties. Within military history, scholars and military personnel most commonly define four generations of modern warfare (Lind et al. 2001), following up the pre-modern battles fought in antiquity and the Middle Ages, ironically beginning with the Peace of Westphalia in 1648 (Reed 2008). According to Lind et al. (2001), the first of these generations consisted mainly of combat in line and column, linearity and military drill originated here, especially important in the era of the smoothbore musket, to maximize firepower. The authors continue that the coming of the second generation was marked by the invention of the rifled musket, breech-loaders, barbed wire, and particularly indirect fire, putting emphasis on the fact that during the era of industrial revolution, massed firepower and technology replaced manpower, in accordance with the French motto “the artillery conquers, the infantry occupies” (Lind et al. 2001). While development of new technologies (e.g. armored units like tanks) also factors in, drivers of the third generation of warfare were primarily new ideas and strategies: At the operational level, fresh tactics and maneuvers could prove essential for victory in battle, exemplified for instance by German tactics in World War I (Reed 2008) and the ‘Blitzkrieg’ (Lind et al. 2001). Lind et al. continue to develop the idea of a fourth generation of war, in which decentralization plays a big part, wars will be “widely dispersed and largely undefined”, “nonlinear”, even further relying on outmaneuvering the enemy instead of beating them by sheer mass of forces, with the distinction of military and civilian being blurred, and sometimes without clearly discernible battlefields. Elements of fourth-generation warfare can also be found in guerilla tactics (Hammes 2005) and modern terrorism (van der Klaauw 2021).

Others have expanded this generational concept to include five (Reed 2008) or even six generations (van der Klaauw 2021; McDermott 2021), and terms like aforementioned “post-modern war” (Duffield 1998), “new war” (Kaldor 2012), or “hybrid war” (Hoffman 2007) have been coined to describe contemporary conflicts. What all these have in common is that they refer to a type of confrontation in which there is no clear distinction between private and public, state and non-state, local and global warfare, and organized crime and human rights violations in general, compared to an “old war” in which only the combat between two parties would be decisive (Kaldor 2012)—thus further expanding the dispersion already seen in the fourth generation, with strategies now also including disinformation tactics, covert operations, and cyber-attacks.

Be that as it may, and as shall be examined closer below, there is one constant throughout military history, even though warfare itself, ideas, tactics, and techniques employed differ radically: Infectious diseases are a considerable threat to the health of all parties involved, even in “post-modern wars”. This article may thus aid in raising awareness for these—and especially for malaria—during times of unrest for both clinicians and researchers concerning themselves with public health, military history, the ecology of diseases, biosecurity, and conflict studies.

## Antiquity

One of the first descriptions of malaria and thus also one of the first descriptions of its pathognomonic paroxysms, consisting of chills, followed by fever, then sweats and exacerbation, stems from ancient Greek physician Hippocrates of Kos (460–370 bc) in the first volume of his ‘Of the Epidemics’ (Cunha and Cunha 2008; Boualam et al. 2021).^3^

During his times, πυρετóζ, meaning ‘fever’, chiefly referred to malaria.

Shortly after Hippocrates’ death, one of the arguably most famous military leaders in all of world history was born: Alexander III of Macedonia, better known as Alexander the Great (356–323 bc). Having never lost a battle as a commander, he built one of the biggest empires ever to exist through perhaps unrivaled military genius. His untimely death at age 33 in Babylon prevented him from conquering even further regions and marks the beginning of the Wars of the Diadochi and the subsequent Hellenistic period. Regarding the happenings around his demise, Alexander began to feel feverish in the beginning of June 323 bc; his condition worsened with the fever leading to his death roughly 10 days later (Cunha 2004). Speculations about the cause of his death have included non-infectious ones, like acute pancreatitis (Sbarounis 1997), endocarditis, Guillain–Barré syndrome (Hall 2018), and poisoning with various possible substances,^4^ or infectious diseases such as West Nile fever and influenza; however, due to the description of the feverish illness, both typhoid fever and malaria are highly probable contenders to be his killers (Cunha 2004), and scholars are divided on this issue. Nevertheless, there is evidence in the ancient text that the fever was intermittent, which is highly suggestive of *Plasmodium* infection (Sallares 2002a), with malaria thus halting one of the most successful military campaigns in world history due to the loss of the critical figurehead of the Macedonian army.

Apart from Ancient Greece, malaria also had a great influence on the Roman Empire, where it was known as *febris ardens*, which can be translated with ‘intense burning heat’, and believed to be caused by *mal aria* (‘bad air’) (Cunha and Cunha 2008).^5^ Among other things, malaria may have had a contribution in the Empire’s final crumbling (Bray 2004; Lalchhandama 2014), and its influence on the life in the city itself cannot be overstated. This is exemplified by another of antiquity’s most famous physicians, Galen, for the most part practicing in Rome, being intimately familiar with intermittent ‘fevers’, which were most likely malarial (Sallares 2002b).^6^ Ancient historian Kyle Harper calls the illness a “pall over the city” and one of the “main drivers of epidemic mortality in ancient Rome”, with a high prevalence in central and Southern Italy due to its wetland ecology and a striking increase of mortality in the late summer and autumn months, fitting well with a rise in seasonal *P. falciparum* infections (Harper 2017a). Interestingly, the first ‘barbarian’ conqueror of Rome is another military leader whose life and campaign were cut short most likely due to *P. falciparum* malaria (Galassi et al. 2016; Faure 2017). Alaric I (ca. 370–410 ad), king of the Visigoths, had sacked Rome in 410; on return from the conquest, it is supposed that he contracted malaria either in the city itself or in the Pontine Marshes.

On the other hand, the city of Rome itself was later saved from another sack thanks to the illness: analysis of DNA is suggesting that Atilla the Hun abandoned his conquest of Italy in 452 ad after raiding the northern part of the country en route to Rome because he was afraid of his army’s exposure to malaria and not because of the appeasement talks by Pope Leo (Carroll 2001).

Malaria incidence characteristically increased during the summer months in Italy; this was felt especially by the troops of the Byzantian general Flavius Belisarius, when in 536 ad, he laid siege on Rome itself, intending to starve out the city (Ayoade 2017). In the surrounding Campagna, they dug out entrenchments, which soon led to the decimation of the soldiers’ numbers due to bites by infected mosquitoes, forcing Belisarius, who had contracted malaria himself (Sallares 2002c), to abandon his objective.

It can generally be said that artificial changes in ecosystems made during wars, such as mentioned trench and road constructions, the destruction of dams (Shah 2010a), logging of forests, and cessation of field tillage accelerate the spread of the disease, often because still bodies of waters are created, resulting in excellent mosquito breeding sites. Likewise, population movements, which naturally occur during wartime, may introduce other parasite strains into human communities that are immunologically naïve to the specific *Plasmodium* strain or *Plasmodium* in general (ibid.). Chronic malnutrition, often experienced by soldiers and civilians alike during times of conflict, may aggravate *Plasmodium* infections (Das et al. 2018) and is another point to consider here. This all is exemplified in the Roman countryside, which became highly malarious due to an “interplay of war, politics and changing patterns of land use”, as historian Randall M. Packard puts it, “altering the distribution of water, creating anopheline breeding grounds, introducing malaria parasites, and exposing local populations to malarial infections” (Packard 2007b).

## The Middle Ages

On the other hand, Rome experienced protection against a multitude of enemies due to malaria, for instance from the Carthaginians under Hannibal, the aforementioned Visigoths under Alaric, the Huns under Atilla (Harper 2017b), and the Vandals under Geiseric (Winegard 2019a).

The illness, also known as the “air of Rome” (Celli-Fraentzel 1932) in the Middle Ages, moreover interfered with the military campaigns of many a Holy Roman Emperor: this pertains to Otto I, Otto II, Heinrich II, Heinrich IV, and Friedrich I Barbarossa. Otto I aborted his pacification mission of Italy in 1022 due to the infections in his army; Heinrich IV, on the other hand, besieged Rome four times between 1081 and 1084, never being able to actually conquer the city as he had to withdraw his forces from the mosquito-ridden Campagna each time. Their successor Friedrich I, after ending the siege of Ancona, marched on and conquered Rome in 1167, where he was crowned emperor (Comyn 1841). He had barely established himself in the city, when a ‘pestilence’ began to run rampant within his army, claiming also the lives of notable bishops and noblemen in his retinue. Involuntarily, Barbarossa had to retreat back across the Alps, supposedly the cause for the deaths was none other than malaria (Opll 1987; Herde 1991; Pohl 2017). Thirty years later, Barbarossa’s son, Holy Roman Emperor Heinrich VI, allegedly also died of a malaria infection (Celli-Fraentzel 1932). Overall, the Pontine Marshes, prime mosquito breeding grounds southeast of Rome, thus sheltered the city and also the Vatican from foreign invasion.

There is a multitude of well-known medieval malaria victims, maybe so because their cause of death is more likely to be recorded if compared to the simple peasant: Other “crowned heads” (Celli-Fraentzel 1932), many “church officials” (Winegard 2019a), among them four popes in only one century (Rocco 2003), and famous poet Dante Alighieri (Raffa 2020) are thought to have fallen victim to the parasitosis, to just name a few.

During the First (1096–1099), Second (1147–1149), and Third (1188–1192) Crusade, all of the crusaders’ troops experienced massive losses due to malaria, with it killing 35 percent of European soldiers in the first three years of the Third Crusade (Winegard 2019a). The strategic importance of the disease cannot be overstated for these campaigns, with the Muslim defenders having acquired both immunity to local *Plasmodium* strains and genetic characteristics preventing or impeding infection, for instance Duffy blood group negativity.

Further to the east, Genghis Khan’s ambitions to conquer Eastern Europe were halted in 1241, when his army was decimated due to a wet spring in the Hungarian plains; his death is suspected to be caused by an immune deficiency after multiple bouts of the illness (Winegard 2019b). Genghis’ grandson Kublai’s military efforts in Europe, the Levant, and continental East Asia all were greatly attenuated by malaria as well (ibid.).

## The Modern Era

Some scholars base the beginning of the modern period on Christopher Columbus’ voyages to the Americas in 1492. While there is some evidence for malaria infection in South American mummies before his journeys (Rodrigues et al. 2018), the European invasion of the continents in the sixteenth century introduced the parasitosis in the Americas on an extreme scale (de Castro and Singer 2005; Yalcindag et al. 2012), assisting—among other diseases—the Europeans in overwhelming the indigenous peoples.

A few hundred years later, during the American Revolutionary War (1775–1783), malaria infections among the British troops were so severe that half of the army was unable to move while stationed in the malarious South Carolina low country in the summer of 1780 (McCandless 2007; McNeill 2016). Historian Peter McCandless argues that “disease, particularly malaria, reduced British fighting capacity more effectively than patriot bullets” (McCandless 2007). The American adversaries in the South had meanwhile largely grown up with the disease, rendering them at least partially immune.^7^ In the following year, Lord Cornwallis, the British commander, relocated the troops further north for evading the “fatal sickness which so nearly ruined the army”; his supervisors wanted him to base himself at Yorktown, where malaria began to run rampant again in the summer months, leading to 51 percent of his army not being able to stand (McNeill 2016). In turn, French and American troops prevented him from moving his soldiers, resulting in Cornwallis’ surrender in October of the same year (ibid.).

In 1791, when more than 100,000 slaves in Haiti revolted against the French plantation owners, the British entertained the notion that this uprising might become a template for similar events in their own Caribbean dependencies and therefore decided to intervene two years later. Over the next five years, 15,000 of the 23,000 British soldiers deployed to Hispaniola died of either malaria or yellow fever,^8^ until the British eventually withdrew (Winegard 2019c). Similarly, Napoleon’s subsequent mission in Haiti shortly afterward saw 55,000 out of 65,000 French soldiers that were deployed die of mosquito-borne diseases. Both the British and French interventions on the island were thus severely hampered not only by malaria, but also by yellow fever (Perry 1996), an illness also transmitted via mosquito bite and caused by a flavivirus. Here, it must be taken into account that for contemporaries—and even more when studying the numbers in historical records retrospectively—it may prove nearly impossible to distinguish between malaria and yellow fever in some cases.

Roughly 10 years later, in Europe, the British launched a military campaign to establish another front against Napoleon by destroying the French fleet and arsenals in Holland and to make the river Scheldt inaccessible to France (Lynch 2009). Thus, 42,000 British soldiers landed on Walcheren Island in the Scheldt estuary in July 1809. Soon, these soldiers were afflicted with an illness later known as ‘Walcheren fever’, today thought to be most likely a combination of malaria, typhus, typhoid fever, and possibly dysentery (Howard 1999). Fighting was sporadic and eventually, when the campaign was ended in February 1810, only 100 men had died in combat (showing the military irrelevancy of the expedition), while the fever took the lives of 3,900 soldiers with over 40 percent of the army having been feverish and 11,000 men still reporting ill. Malaria transmission was increased by the fact that Napoleon had his soldiers break dikes and flood the already swampy area with brackish water; he can be quoted with “We must oppose the English with nothing but fever, which will soon devour them all” (Winegard 2019c).

The course of the Civil War in America (1861–1865) was also distinctly shaped by malaria, or as military surgeon E. C. Bidwell put it: “The subtle malaria of the rebel soil destroys and disables more Northern soldiers than all the wounds received from rebel arms” (Bell 2010). Unlike in the Revolutionary War, malaria did not sway the course of the conflict to one side, but rather inflicted damage on all belligerents (Athni et al. 2021). Sixty-six percent of deaths during this war in the US army were due to disease rather than fighting, similar numbers can be assumed for the Confederate army, with malaria being the dominant cause. Between 1861 and 1865 a staggering 1,315,955 cases of the parasitosis were diagnosed in Union troops, apart from critically hampering war efforts also accounting for 10,000 (Sartin 1993), or about 10 percent of total deaths (Packard 2007a). Over half of all troops in the Civil War acquired malaria (Prinzing 1916). Other sources give an incidence rate of 2,698 cases per 1,000 troops in the same timeframe in Union soldiers, and 41,539 cases in 18 months from January 1862 to July 1863 (Packard 2007c). Infectious diseases, of which malaria was a prominent representative, accounted for roughly two thirds of all 660,000 soldier’s deaths, impeding major campaigns; it is surmised that this extended the fighting by up to two years (Sartin 1993). Malaria also spread in the civilian population in previously non-endemic areas after both the American Revolutionary and Civil War (Packard 2007a).

In addition, malaria epidemics could frequently be encountered on US and Royal Navy Warships in the nineteenth century, particularly when these were on antislavery patrol close to the African Coast; these warship-board malaria outbreaks would continue well into the twentieth century, even after the antimalarial quinine became available (Shanks 2021).

Industrialization brought with itself relatively poorer health coverage and increased numbers of soldiers in war. Therefore, many more examples of malaria having a crucial impact on the outcome of wars can be found over the late modern period, for instance Napoleon’s siege of Mantua (1796–1797), where the French General allegedly flooded the plains to facilitate the spread of malaria (Frischknecht 2003), during the Second Italian War of Independence (1859) (Councell 1941) or the French conquest of Madagascar (1895) with 6,000 malaria-related deaths, whereas only 30 combatants were killed in action on the island (Migliani et al. 2014).

## A Short Digression on Malaria Distribution and Elimination in Europe

As mentioned before, malaria endemicity was significantly more widespread, reaching its maximal pre-intervention distribution around 1900, stretching from the 64th parallel north to the 32nd parallel south, which corresponds roughly to the 15 °C July and January isotherms (Hay et al. 2004) (see also Fig. 1). Here, *P. vivax* transmission would still be possible (e.g., also in the more temperate regions of Europe), whereas modelings have shown a more limited temperature suitability for *P. falciparum* (Gething et al. 2011), confining the latter to a slightly smaller area around the equator. Several species of the *Anopheles* vector existed in Europe. It is supposed that *Plasmodium* first reached the Mediterranean in the Neolithic, spreading to the rest of the continent (Piperaki 2018). As *P. malariae* and *P. vivax* made their way into Northwestern Europe, *P. falciparum* was rarely found in the North, also due to poor adaptation to local mosquito species (ibid.) and the differences in temperature suitability. The process of malaria elimination from Europe manifested itself from the Northwest to the Southeast and was driven by a multitude of changes, among them environmental, ecological, social, and treatment developments as well as coordinated control measures.

For the European continent, the historical efforts to eradicate malaria are fairly well documented, at the onset concentrating mostly on high-risk zones such as swamps and still waters (Boualam et al. 2021). Drainage and reclamation of these swamps and marshlands as well as water control measures limited mosquito breeding. Accordingly, French records show decrees for marsh drainages from the year 1599 onward (ibid.). Another important aspect in the eventual eradication might have been the segregation in habitat between humans and livestock and the overall higher cattle population (Piperaki 2018): In Great Britain, an investigation has shown receding acreages of wetlands and an increase in the rearing of cattle to both independently play a role in the malaria incidence decline between the years 1810 and 1940, by respectively limiting *Anopheles* breeding grounds and perhaps shifting biting behavior of mosquitos from humans to livestock (Kuhn et al. 2003). The authors surmise that 20 percent of the decrease in malaria is explainable by these nonclimatic variables (ibid.). The types of constructions were humans and cattle lived in one area continued to disappear, with the main (zoophilic) European vector, *An. atroparvus*, thus more likely to be found in stables or barns, where animals were kept, instead of human living quarters (Piperaki 2018). Moreover, the progressive introduction of remedies against malaria, for instance when the chinchona bark was brought into Europe in the sixteenth century or quinine became available during the nineteenth century, diminished transmission (ibid.).

In the first half of the twentieth century, international efforts to eradicate malaria were intensified, while improvements in public health services took place, leading to a continuous decline in malaria numbers (Majori 2012). A pioneering example is Italy, as indicated before among the countries of Europe that were suffering the most under the parasitosis. Two million inhabitants were infected or re-infected annually around the turn of the nineteenth to the twentieth century and 11 million out of a population of 25 million were permanently at risk for infection (Snowden 2006b). The economic burden exerted by the disease, especially in the South, often uncoupled from the economic progress made in the more prosperous North, was enormous (ibid.). When Italian public health authorities realized the size of the problem, a national eradication campaign was set up in 1900 (Snowden 2006c), a worldwide first, coinciding with key discoveries in malariology, for instance, the discovery of the *Plasmodium* parasite and *Anopheles* as a vector. As Italian scientists made important contributions to the field, the country’s politicians made crucial decisions by instigating the free production and distribution of quinine and establishing measures to limit mosquito breeding grounds (Majori 2012). Other factors also came into play here: The social and infrastructure changed drastically throughout the first 50 years of the twentieth century. For instance, the city of Rome, a malaria hotbed since antiquity, lost many of its (irrigated) gardens in its transformation into a modern city and thus the mosquito was deprived of breeding sites as well as hiding places (Sallares 2002b). Just as critical for the city was the draining of the Pontine Marshes, an endeavour that had not produced the desired results in ancient Rome, as the drainage scheme proved ineffective because the gradient was not great enough, i.e. the water in the channels became stagnant and overgrown, instead facilitating larvae development—but proved successful in the twentieth century (Sallares 2002c).

With the fascist rise to power, malaria control became a priority once again, when the Department of Health in conjunction with the *Opera Nazionale Combattenti*^9^ declaring a policy called *bonificia integrale*, with a three-stage plan (Snowden 2006d): First, *bonificia idrauliga* (‘hydraulic reclamation’), then *bonificia agraria* (‘agrarian reclamation’) and lastly *bonificia igienica* (‘hygienic reclamation’). The main target for the first stage was the drainage of the swamp, while the second was for marshlands to be settled by homesteads and public infrastructure. The third stage concerned itself mostly with *Anopheles* control measures, quinine distribution, and public health services (ibid.). After trials at the Tiber River, work was started under leadership of Benito Mussolini on the Pontine Marshes in 1929, with the project declared complete a decade later. During this time, the countrywide eradication program was supported by the Rockefeller Foundation, with American scientist L. W. Hackett spearheading, leading to the creation of the ‘Experimental Station for Malaria Control in Italy’, which transformed into the country’s National Institute of Public Health in 1934 (Majori 2012). This Italian eradication campaign faced numerous difficulties, especially because of the World Wars, but was nonetheless continued for more than half a century. Ultimately, it was successful, as Italy was declared malaria-free in 1965, also due to the use of the insecticide dichlorodiphenyltrichloroethane (DDT) after WWII (Snowden 2006c). The European region as defined by the World Health Organization (WHO)^10^ followed suit in 1975 when autochthonous malaria transmission was considered eliminated, except for Turkey (Zhao et al. 2016; Piperaki 2018). Since then, sporadic cases of local malaria transmission have occurred in a handful of European countries, mostly due to infected travelers, migrants, and other population movements and continue to do so until today, even if in 2015 the WHO reported zero indigenous malaria cases in Europe for the first time (Piperaki 2018).

Worldwide, the WHO initiated the ‘Global Eradication of Malaria Program’ in 1955, heavily based on insecticides, particularly indoor spraying with DDT, as well as antimalarials (Majori 2012). Within two decades, malaria elimination was proclaimed for all formerly endemic developed countries, with the scheme also achieving success in significantly moderating transmission in tropical Asia and Latin America (ibid.). On the African continent, the program could not be realized to the same extent and upcoming resistances against insecticides and antimalarials further complicated the situation. As new cases re-emerged in previously malaria-free declared areas, the WHO abandoned the elimination strategy in 1969 and instead focused on control of morbidity and mortality (ibid.). Today, despite all efforts, especially sub-Saharan Africa still suffers severely under the disease, with socioeconomical, ecological, political, and conflict factors once again coming into play here. Particularly the latter, i.e. warfare, thus has many times impeded on control efforts or significantly prolonged the time required to reach elimination in certain areas.

## The First Half of the Twentieth Century and the World Wars

The Franco-Prussian war in 1870 and the Russo-Japanese war of 1904 were the first altercations where infections allegedly killed less combatants than bullets (Councell 1941). For US troops, this came only with World War I (1914–1918) (ibid.). During WWI, militaries greatly underestimated the size of the problem that malaria would pose (Brabin 2014). Military surveillance statistics indicate that at least 1.5 million soldiers were infected, with a case fatality rate of 0.2 to 5 percent depending on location and major epidemics taking place in Palestine, Mesopotamia, Italy, and Macedonia. Especially in the latter of these countries one of the “most notorious malaria epidemics associated with ecological disruptions and wartime population movements” took place (Shah 2010a).

Similarly, Italian public health records show an impressive resurgence in malaria numbers during WWI. Between 1900 and 1914, malaria deaths had steadily dropped from 490 to 57 per million; four years later, the numbers had risen to 325 deaths per million again (Snowden 2006a). While numbers also increased in the civilian population, regarding the situation in the military, geographical factors came into play here: The clashes between Austro-Hungarian and Italian forces in the trenches of the Veneto plains in northern Italy were thus fought in prime mosquito habitat, with the campaigning mostly coinciding with the malaria season, often extending into a time of the day when *Anopheles* is most active, i.e. dusk and dawn; because of these horrendous circumstances, Italy’s Third was called the “malarial army” (ibid.).

In Greece, the German, French, and British armies were immobilized for three years because of malaria infections (at one point a French general excused himself with “regret that my army is in hospital with malaria”) with the French experiencing less severe outcomes due to better quinine prophylaxis (Russel 1963; Brabin 2014). Still, almost 80 percent of 120,000 Frenchmen were hospitalized with malaria; even worse, there were 162,512 hospital admissions for 124,000 British soldiers between 1916 and 1918.

Sir Ronald Ross was in the area in 1917/1918, and as consultant physician to the Mediterranean Expeditionary forces recommended quinine prophylaxis as well, he had in 1897 famously discovered the role of the mosquito as a vector of malaria. Another military physician who made invaluable contributions to malariology was Charles Laveran, who discovered the protozoan parasite responsible in 1880, at a military hospital in Algeria (Bruce-Chwatt 1981).

Regarding the second World War, General Douglas McArthur can be quoted with “this will be a long war if for every division I have facing the enemy I must count on a second division in the hospital with malaria and a third division convalescing from this debilitating disease” (Beaumier et al. 2013). Malaria played a critical role in the tropics during this time, especially the Pacific war theatre, with a staggering number of 572,950 cases for US troops alone (Mackie 1947). In this way, the disease pressured the USA to capitulate at Bataan in the Philippines and Japan to withdraw its forces from Guadalcanal, largest of the Solomon Islands (Joy 1999). Consequently, the antimalarial quinine was fast becoming an extremely valuable resource in WWII (Shah 2010b). Moreover, in 1942, the ‘Malaria Control in War Areas’ (MCWA) program was established by the US Public Health Service for protection of military bases in the Southern United States and Caribbean overseas areas (Parascandola 1996); these measures should later develop into the Centers for Disease Control and Prevention (CDC).

Both WWI and WWII nullified many years of abovementioned control and eradication struggles (Snowden 2006a; Athni et al. 2021). Military personnel weaponized malaria ecology deliberately, at least in part, to cause the maximum of damage both in civilians and soldiers (Athni et al. 2021). There is evidence that research was conducted by Nazi Germany into harnessing this arthropod-borne disease as a biological weapon: at Dachau concentration camp, prisoners were inoculated with malaria and different *Anopheles* species were compared regarding their survivability in a previously created ‘entomological institute’ (Reinhardt 2013). Plans may have also been made to set free infected mosquitoes from airplanes; some historians have entertained the notion based on Allied and Italian records that Germany re-flooded the already mentioned and previously drained Pontine Marshes and then re-introduced infected *Anopheles* there (Vergano 2014).

## The Latter Half of the Twentieth Century and Recent Conflicts

Supposedly, worldwide resurgences in malaria incidence since the 1960s have been facilitated by a triad of political unrest, armed conflict, and population displacement, with disease spikes being associated with almost all bigger wars of the nineteenth and twentieth century (Packard 2007a). Another key factor is poverty, to which malaria is most closely related, bringing with itself both a higher malaria incidence and reciprocally is caused by higher malaria numbers (Teklehaimanot and Mejia 2008). Moreover, many countries in which wars can be observed today have weaker economies, a complex amalgamation of adverse circumstances due to colonialism (leading to a outdistanced position on the world market), inexperienced governance or development agencies, and corruption. On the other hand, war is of course very costly because of a multitude of factors, either further degrading the already debilitated economy or causing a country with a previously reasonably healthy one to go down a slippery slope of recession for the first time.

Some sources claim that more casualties were caused by malaria than by bullets during each US-American military campaign in the twentieth century (PATH’s Malaria Vaccine Initiative 2004). In the Korean (1950–1953) and Vietnam (1962–1975) Wars, there were approximately 35,000 and 65,000 hospital admissions among US soldiers respectively (Beaumier et al. 2013), with the illness posing a far greater threat during the latter conflict as the more pernicious *P. falciparum* was the dominant infecting species, compared to *P. vivax* in Korea (Beadle and Hoffman 1993). The People’s Army of Vietnam and the Viet Cong also suffered increasing morbidity due to malaria; in some locations, combat strength was reduced by half or even up to ninety percent in some cases (Weiyuan 2009).

Advances in many fields of science happen during times of war; the same holds true for developments in malariology. As WWII brought about the widespread use of the antimalarial chloroquine and the important insecticide DDT (Hays 2000; Packard 2007d), of which the structure was already known, but whose insecticidal properties were only discovered in 1939 (Nosten et al. 2022), Project 523 was created by the People’s Republic of China in 1967 to support the Vietnamese military, which ultimately led to the development of today’s mainstay of treatment, the artemisinins (Tu 2011). While this may be a minor upside, it is evident that wars in general undermine public health systems, particularly grievous in the case of malaria, where control programs are of the essence and are known to be prevented from working effectively (Packard 2007a). In the second half of the twentieth century, numerous poignant examples can be found for this matter.^11^

The UN-backed Operation ‘Restore Hope’ (1992–1994) saw malaria as the preeminent cause for casualties with 48 cases diagnosed during deployment and 83 cases after the return from Somalia in US soldiers, while the Iraq War had soldiers often not taking breaks from walking to avoid being bitten by mosquitoes (PATH’s Malaria Vaccine Initiative 2004). Investigative journalist Soniah Shah points out that “in 1996, military troops in war-torn Afghanistan sparked a malaria epidemic across Central Asia” and “[s]oon, Azerbaijan, Tajikistan, and Turkey suffered malaria outbreaks” (Shah 2010c). Another *P. falciparum* malaria wave in Afghanistan during the US military campaign of 2001 claimed 53 lives; of the 157 troops who spent at least one night on land in the US Liberia deployment of 2003, 69 contracted the disease, with a third of all marines sent as military advisors becoming infected (PATH’s Malaria Vaccine Initiative 2004).

In the Democratic Republic of Congo, the civil and international conflict that has been boiling there for decades had cost 3.9 million lives until 2009, but most of these deaths were not attributable to direct violence but rather due to malaria and other preventable infectious diseases (Hawkes et al. 2009). In this country, the public health system costs of warfare became obvious in the 1990s, where access to health services decreased by 70 percent in that timeframe (Packard 2007a).

Very recently, epidemiological analyses saw extremely high rates of both *P. vivax* and *P. falciparum* malaria in children in the war-torn Orakzai region of Pakistan accompanying a public health system breakdown (Karim et al. 2016); similarly, in the conflict zone of South Sudan, elevated rates of both malnutrition and malaria infection were found in children and adolescents, with a prevalence of infection in up to 40 percent of individuals (Charchuk et al. 2015). Much alike, the war that boils in the Tigray region of Ethiopia since late 2020 has brought the health system there to a collapse, with a 2021 study highlighting the increased likelihood of malaria outbreaks because of this (Gesesew et al. 2021).

Another important consequence of warfare, only mentioned in short before, is human displacement, with one of the main culprits of morbidity and mortality in refugee camps today being malaria (Anderson et al. 2011). The high prevalence of malaria in refugee populations is facilitated by the conditions within these camps: often parasites are introduced in immunologically naïve populations, which are moreover of high vulnerability because of predisposing factors like malnourishment, anemia, concomitant infections, a crowded setting lack, of malaria control measures, healthcare workers, and adequate treatment (World Health Organization 2013; Messenger et al. 2021). These conditions expedite transmission within the camps and surrounding areas especially following warfare (Packard 2007a). Hence, many examples of malaria outbreaks within refugee populations abound (ibid., Shanks 2023).

In the aftermath of WWI, the Greco-Turkish War in Asia Minor ensued, accompanied by a refugee crisis with 1.3 million displaced Greeks, with an extensive epidemic and a high death toll occurring (Piperaki 2018). When more than two million Afghans were forced to flee into Pakistan in the late 1970s and early 1980s, there was a significantly higher number of infections among the refugees than the local population (Suleman 1988). Although case numbers also rose in the Pakistani populace because of the influx of refugees (Packard 2007a), they increased much quicker in the Afghan camps, probably because of low herd immunity (Suleman 1988). The disease was the leading cause of death in displaced persons from Cambodia after they had to traverse zones with malaria endemicity bound for camps in Eastern Thailand in 1979 (Rowland and Nosten 2001), while there were significantly less malarious camps with individuals who trekked only through malaria-free areas via a more northern route (Hurwitz 1979). Five years later, once again in Thailand, an incidence rate of 1,037 per 1,000 was found, this time among Myanmar refugees; the overwhelming majority were cases due to *P. falciparum* infection (Bloland et al. 2002). Likewise, in the early 1990s, malaria was the principal cause of death in adult refugees from Mozambique in Malawi and in Ethiopian refugees in Sudan, as well as the second most common during the refugee crisis in Zaire (now the Democratic Republic of Congo) in 1994, even amidst an enormous cholera and dysentery epidemic (ibid.). The same problem among displaced populations continued well into the 2000s, especially on the African continent, and up until today.

With numbers of refugees steadily climbing, the risk of malaria outbreaks with the possibility of (re-)introducing the disease in previously non-endemic regions is only going to increase.^12^ As of 2022, 108.4 million people were “forcibly displaced as a result of persecution, conflict, violence, human rights violations and events seriously disturbing public order” according to the United Nations Refugee Agency, with numbers having more than doubled since 2012 (UNHCR 2023), a time when already two thirds of these were living in malaria-endemic areas of the world (Anderson et al. 2011). If mapped out today, the distribution of people living in humanitarian emergency settings matches the *P. falciparum* parasite rate in children aged two to ten—one of the groups with the highest mortality risk due to severe malaria—to a strong degree, with sub-Saharan Africa being a hot spot (Messenger et al. 2021).

## Conclusion

This article encompasses and compresses thousands of years of human history. Therefore, it may only paint the picture of the connection between infectious diseases and armed conflicts in broad strokes, especially when one factors in that the lion’s share of events summarized are mostly part of ‘Western’ history. However, this longitudinal approach was chosen to underline the lasting importance of infectious diseases, especially malaria, in the history of warfare, from antiquity until recent times, well into the ‘post-modern’ era. This critical role becomes apparent by malaria mortality often outnumbering the death toll of the actual direct violence of the conflict and greatly aggravating other public health problems, and vice versa, as well as critically impeding control measures and eradication efforts. Warfare hence comes with conditions under which infectious and vector-borne diseases thrive. Contemporary clinicians must consequently be aware of the possibility of a resurgence of malaria incidence in emerging zones of combat and displaced human populations.

Nevertheless, there is also a glimmer of hope on the horizon regarding malaria and conflict: it comes in the form of the nations of El Salvador and Sri Lanka. In the former, an eradication program had proven very successful amidst civil conflicts in the 1980s (Packard 2007a), whereas in Sri Lanka, malaria elimination was officially declared by the WHO fairly recently in 2016, despite the country having suffered from a 26-year-long civil war (Ahmed et al. 2021). Strategies employed there could serve as a template for future elimination campaigns in other war-ravaged regions with malaria endemicity such as Somalia, Ethiopia, Yemen, and Afghanistan.

## Data Availability

No datasets were generated or analysed during the current study.

## Footnotes

1 Genetic adaptations like the sickle-cell trait (Williams et al. 2005), thalassemia or glucose-6-phosphate dehydrogenase deficiency confer resistance against malaria and therefore a selective advantage in endemic areas, outweighing the risk for potential disadvantageous consequences (Brown 1986). On the other hand, humans have also adapted their behaviors to lower the risk of contracting the disease. On the malaria-endemic island of Sardinia, where thalassemia and glucose-6-phosphate dehydrogenase deficiency are encountered frequently, pastoralism (i.e. the grazing of livestock) follows an inverse pattern of transhumance, meaning that permanent settlements are located in the highlands and flocks are taken to a lower altitude in the winter (Brown 1986). While this brings with itself disadvantages in production efficiency, it is overshadowed by the avoidance of the lowlands during the summer malaria season. Especially in Italy, more examples can be found for movement patterns marked by the avoidance of malarious areas during the summer months: There were seasonal constraints on iron production in the Maremma, Tuscany during the nineteenth century, where blast furnaces were only being used for six to ten months from the end of summer to the beginning of the following spring, with the workers both in metal production as well as agriculture arriving in settlements in late summer (Boldrini 2006). More evidence of abandonment during the summer can be found for other urban settlements in the region, for instance in the town of Follonica (De Leo, personal communication, 15 Feb 2024). Moreover, women in aforementioned Sardinian lowland settlements were traditionally limited in their geographical mobility, being discouraged from working in the fields and rather staying at home, particularly when pregnant or having just given birth, thus restricting their exposure to the anopheline vector (Brown 1986). According to some scholars, gender-specific movement patterns confining females from accessing swampy areas also existed in ancient Rome (Athni et al. 2021), likely established to avert malaria during pregnancy, which frequently lead to adverse birth outcomes and miscarriages (Stivala 2015), also endangering the life of the mother because of uncontrollable hemorrhages (Phang 2022). These interesting contributions are to be considered carefully though, as restriction of women’s movements and particularly of pregnant women, to allegedly protect them from malaria, may also intersect with the oppressive norms of a patriarchal society (De Leo, personal communication, 15 Feb 2024). Anthropologist Peter J. Brown argues that behavioral adaptations work in a preventive sense compared to the more therapeutic (i.e., mortality-reducing once the disease is contracted) genetic adaptations, maybe at an even lower cost (loss of production efficiency); meanwhile, carriers of a homozygous mutation of the recessive thalassemia trait experience severe anemia, deformities of the face, and usually death at an early age (‘thalassaemia major’), while glucose-6-phosphate dehydrogenase–deficient individuals may have to cope with favism (Brown 1986).

2 Malaria was of such (cultural) significance that the ‘agues’, the contemporary English term, or descriptions of it, can be encountered in Geoffrey Chaucer’s ‘Canterbury Tales’ as well as several of William Shakespeare’s plays and poems (Reiter 2000; Boualam et al. 2021).

3 Translated from this work, the Hippocratic description goes as follows (Cunha and Cunha 2008): “When the paroxysms fall on even days, the crises will be on even days; and when the paroxysms fall on odd days, the crises will be on odd days. Thus, the first interval of those with crises on even days is on the fourth day, the sixth day, the eighth day, the tenth day, the fourteenth day, the twentieth day, the twenty fourth day, the thirtieth day, the fortieth day, the sixtieth day, the eightieth day, and the one hundred and twentieth day. While those with crises on odd days, the first interval is on the third day, the fifth day, the seventh day, the ninth day, the eleventh day, the seventeenth day, the twenty-first day, the twenty-seventh day, and the thirty-first day. Furthermore, it is necessary that one know that if crises fall on days other than those mentioned above, there will be a relapse, and this may be deadly. But it is essential to pay attention and know at which times the crises will lead to death and in which to recovery, or during which is there tendency to fair better or worse. The intervals when crises occur in irregular fevers, quartans, quintans, septans and nonanes, should also be considered.”

4 Many possible toxins have been suggested, among them strychnine, arsenical compounds, lead, belladonna, methanol, and white hellebore (Mishra et al. 2022).

5 It should be mentioned here that it was not before the late nineteenth century that—due to the seminal works of figureheads like John Snow, Louis Pasteur, and Robert Koch—the germ theory of disease was widely accepted. Before that, with it originating at least in part from ideas of aforementioned Hippocrates, the occurrence of illnesses was by and large explained by the miasma theory, i.e. the concept that so-called *miasmas* (‘pollutions’ in Ancient Greek), toxic emissions of foul air, were responsible (Kannadan 2018). The etymology of malaria obviously points to this. Paired with the pre-modern level of medical knowledge, malaria was most likely a ‘broader concept’ rather than a distinct, clinically specified disease.

6 Roberto Sallares, who wrote a seminal work on malaria in ancient Rome, quotes Galen with the following: “That there is such a fever, as I have stated, does not require the evidence of Hippocrates or anyone else as a witness, since we observe it every day, especially at Rome. For just as other diseases are common in other places, so this evil is frequent in this city. […] The fever arising from yellow bile on its own is called tertian, but the fever which requires an equally strong share of the raw humour is strictly called semitertian. It is most frequent at Rome, being very familiar to men in the city.” Tertian fever most likely refers to *P. vivax* infection, while semitertian, much more dangerous according to Galen, pertains to *P. falciparum* (Sallares 2002b).

7 That is, parasites can be observed in the blood (parasitemia), but no clinical symptoms are to be discerned.

8 With these and the following death or disease tolls, one must keep in mind that numbers and causes of casualties, especially before the latter half of the twentieth century, are almost always estimates and harbor an inherent, varying degree of uncertainty. In the majority of cases moreover, malaria mortality numbers are more accurate than those for morbidity (Snowden 2006a).

9 The *ONC* was a charitable organization, providing aid for WWI veterans in Italy.

10 This WHO European region is not equal to Europe in the strict geographical sense, but comprises 53 nations in total, also incorporating countries in the Balkans, South Caucasus, Central Asia, Russia, Turkey, and Iceland (World Health Organization 2023b).

11 In ‘The Making of a Tropical Disease’, the author lists the following examples (Packard 2007a): First, Nicaragua, where malaria control programs were established between 1980 and 1984, saw a 67 percent decline of cases in areas of peace, whereas in war-ridden zones, cases went up by 17 percent (Garfield et al. 1989). Second, the health system collapse following the breaking of the Soviet Union and civil war in Tajikistan facilitated an increase from 616 malaria cases in 1993 to 16,521 in 1996 (Pitt et al. 1998). Third, Burundi, where another civil war forced public health authorities to abandon control measures in the early 1990s, after roughly a decade and vector-favoring weather conditions in the year before, a staggering case number of 3 million in a population of 6.5 million was reported (Checchi et al. 2006).

12 This is exemplified, as Packard once again points out, in the East African Country of Burundi, where refugees from the malaria-endemic lowlands introduced the disease to the previously disease-free highlands over the course of the civil war (Packard 2007a; Protopopoff et al. 2007). See also the Afghan refugee crisis in Pakistan in 1979 mentioned above.
